# Mesenchymal Stromal Cell-Derived Extracellular Vesicles for Oral Mucosal Engraftment in Urethral Reconstruction: Influence of Tissue Origin and Culture Growth Phase (Log vs. Stationary) on miRNA Content

**DOI:** 10.3390/ijms26199412

**Published:** 2025-09-26

**Authors:** Daisuke Watanabe, Akio Mizushima, Akio Horiguchi

**Affiliations:** 1 Department of Molecular and Cellular Therapeutics, Graduate School of Medicine, Juntendo University, Tokyo 113-8421, Japan; akiom@juntendo.ac.jp; 2 Department of Urology, Koto Hospital, Tokyo 136-0072, Japan; 3 Division of Reconstruction, Center for Trauma, Burn and Tactical Medicine, National Defense Medical College Hospital, Saitama 359-8513, Japan; impreza@ndmc.ac.jp

**Keywords:** cell culture growth phase, extracellular vesicles, mesenchymal stromal cells, microRNA, oral mucosal engraftment, urethral stricture, regenerative medicine

## Abstract

Urethral stricture involves fibrotic narrowing of the urethral mucosa and spongiosum. Although urethroplasty using oral mucosal grafts is the gold standard for complex cases due to its high success rate, technical complexity limits its broader adoption. To address this, endoscopic transplantation of oral mucosal tissue has been proposed. While feasibility has been demonstrated, clinical efficacy remains suboptimal. Developing adjunctive factors that facilitate mucosal engraftment may improve outcomes of endoscopic transplantation. Extracellular vesicles (EVs)—membrane-bound nanoparticles secreted by cells that deliver miRNAs and other bioactive molecules—have recently emerged as promising candidates. We investigated EVs derived from four mesenchymal stromal cell (MSC) sources—stem cells from human exfoliated deciduous teeth (SHED), adipose tissue, umbilical cord, and bone marrow (BM)—isolated during both logarithmic (log) and stationary culture phases. miRNA profiling revealed distinct phase- and origin-specific signatures. SHED-derived EVs from the log phase and bone marrow-derived EVs from the stationary phase expressed miR-31, the let-7 family, and miR-205, suggesting early wound healing potential. In contrast, stationary-phase SHED-EVs and log-phase BM-MSC-EVs were enriched in the miR-99 family and miR-31, indicating potential roles in epithelial stabilization and fibrosis modulation. These findings support phase-specific application of MSC-EVs to optimize mucosal engraftment in transurethral reconstruction.

## 1. Introduction

Urethral stricture is a common urological condition that often leads to repeated endoscopic interventions such as dilation or direct visual internal urethrotomy (DVIU), despite the superior outcomes of urethroplasty. Urethroplasty, particularly techniques involving oral mucosal grafts, is considered the gold standard for curative treatment of complex or recurrent urethral strictures [1,2,3]. However, access to urethroplasty remains limited, and patients who can undergo this definitive treatment represent only a fraction of those affected—typically those with the means to travel to specialized centers [4,5,6]. Most patients are managed conservatively, frequently resulting in recurrence and suboptimal outcomes. Although the broader dissemination of urethroplasty is an ideal goal, its widespread implementation faces significant challenges—not only logistical and geographic barriers, but also a shortage of surgeons with specialized expertise in urethral reconstruction [4,5,6].

In contrast, endoscopic treatments such as DVIU are widely performed due to their minimally invasive nature and procedural simplicity, despite limitations in long-term efficacy. This ubiquity presents an opportunity: the therapeutic potential of endoscopic management may be significantly enhanced through the addition of biological adjuncts. Notably, recent studies—including the work of Nikolavsky et al.—have demonstrated the feasibility of transurethral mucosal regeneration by delivering minced buccal mucosa combined with fibrin glue, showing promising results in a rabbit model [7,8]. As preclinical evidence for transurethral oral mucosal grafting continues to accumulate, there is growing interest in enhancing graft take and mucosal proliferation by supplementing with soluble biological factors that support epithelial regeneration and attenuate fibrosis.

Extracellular vesicles (EVs) are nano-sized, membrane-bound particles secreted by virtually all cell types. They function as carriers of bioactive molecules—including proteins, lipids, and nucleic acids such as microRNAs (miRNAs)—and mediate intercellular communication critical for tissue repair and regeneration. Among various EV sources, mesenchymal stromal cell-derived EVs (MSC-EVs) have attracted particular attention due to their immunomodulatory and pro-regenerative properties. Recent studies have shown that EVs derived from different MSC sources—such as bone marrow, adipose tissue, umbilical cord, and dental pulp—exhibit distinct miRNA signatures, each with unique regenerative profiles. For instance, miR-21 and miR-146a, both commonly enriched in MSC-EVs, have been implicated in key regenerative processes: miR-21 is known to promote epithelial cell proliferation and wound healing by modulating pathways such as PTEN/PI3K-Akt, while miR-146a has been shown to suppress oxidative stress-induced senescence and enhance angiogenesis and cell migration in endothelial cells through the downregulation of the Src signaling pathway [9]. Given these properties, MSC-EVs represent a promising acellular therapeutic candidate capable of promoting graft survival and epithelialization in mucosal tissue repair, including transurethral oral mucosal grafting.

In this study, we explored the therapeutic potential of MSC-EVs in the context of mucosal tissue repair. To our knowledge, this is the first comparative analysis to examine EVs collected from multiple MSC sources—specifically bone marrow, adipose tissue, umbilical cord, and dental pulp—at distinct phases of in vitro culture: the logarithmic (log) and stationary phases. Given the increasing clinical interest in utilizing MSC-EVs for tissue regeneration, it is essential to determine the optimal cellular conditions for EV harvest. Our study provides new insights into how the proliferative status of MSCs influences the miRNA cargo of EVs and identifies culture-phase-dependent differences that may impact their efficacy in supporting mucosal epithelial regeneration. These findings may help inform manufacturing strategies for scalable and reproducible EV-based therapeutics.

## 2. Results

### 2.1. EV Yield Across MSC Sources and Growth Phases

EV concentrations quantified by nanoparticle tracking analysis are summarized in Table 1. In the log phase, EV yield was highest in SHED (1.97 × 10^9^ particles/mL), followed by AD-MSC (1.64 × 10^9^), BM-MSC (1.49 × 10^9^), and UC-MSC (1.39 × 10^9^). In the stationary phase, UC-MSC exceeded the other sources (1.54 × 10^9^), whereas SHED and AD-MSC declined to 1.18 × 10^9^ and 1.10 × 10^9^, respectively, and BM-MSC showed the largest decrease to 4.18 × 10^8^. Relative to log-phase levels, stationary-phase yields decreased for SHED (−40%), AD-MSC (−33%), and BM-MSC (−72%), but increased for UC-MSC (+11%), yielding stationary/log ratios of 0.60, 0.67, 0.28, and 1.11, respectively. These data suggest source- and phase-dependent differences in EV secretion dynamics, with UC-MSCs potentially maintaining or slightly increasing production under stationary conditions.

### 2.2. Global miRNA Landscape Across MSC-EV Sources and Growth Phases

**Figure 1:**
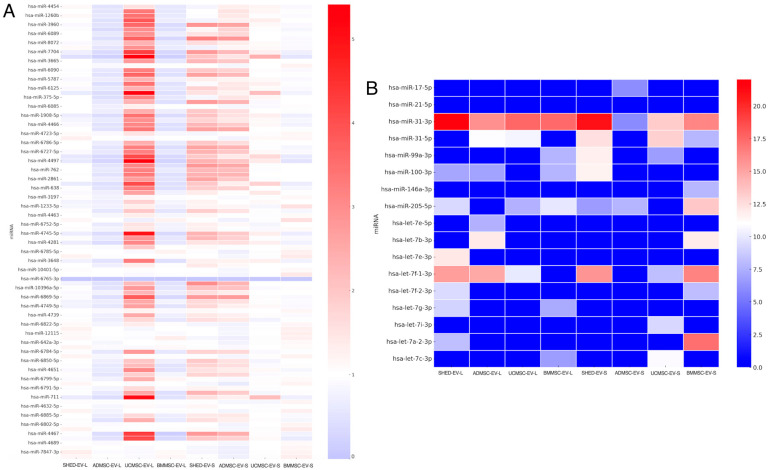
miRNA expression heatmaps of extracellular vesicles (EVs) derived from mesenchymal stromal cells (MSCs) under log and stationary culture phases. (**A**) Heatmap of the top 50 most highly expressed miRNAs across EVs derived from four MSC sources: SHED (stem cells from human exfoliated deciduous teeth), AD-MSC (adipose-derived MSCs), UC-MSC (umbilical cord-derived MSCs), and BM-MSC (bone marrow-derived MSCs). The suffix “-L” indicates EVs collected during the log (exponential growth) phase, while “-S” indicates EVs collected during the stationary (growth plateau) phase. miRNA expression values were normalized and median-centered. Red indicates higher expression relative to the median, and blue indicates lower expression. (**B**) Focused heatmap of miRNAs associated with epithelial regeneration and fibrosis modulation, including miR-31-5p, miR-21-5p, miR-205-5p, miR-99a-3p, miR-100-3p, miR-17-5p, miR-146a-3p, and several let-7 family members.

To compare the global distribution of miRNAs across EVs isolated from different MSC sources, we first generated a heatmap of the top 50 most highly expressed miRNAs (Figure 1A). Notably, UC-MSC-EV-L exhibited the broadest and most intense miRNA expression profile among all conditions, whereas expression levels declined markedly in the stationary phase (UC-MSC-EV-S). In contrast, EVs derived from SHED, AD-MSCs, and BM-MSCs demonstrated globally increased miRNA expression during the stationary phase relative to their log-phase counterparts. These findings highlight distinct tissue- and phase-dependent patterns of miRNA packaging into EVs.

### 2.3. Regeneration- and Fibrosis-Related miRNAs Show Source- and Phase-Specific Enrichment

We next focused on selected miRNAs previously implicated in epithelial regeneration and fibrosis modulation (Figure 1B). Among these, miR-31-3p was most highly expressed in both SHED-EV-L and SHED-EV-S, with moderate levels also observed in UC-MSC-EV-L and BM-MSC-EV-L. miR-31-5p showed relatively higher expression in SHED-EV-S compared to SHED-EV-L. Within the miR-99 family, miR-99a-3p and miR-100-3p were most enriched in SHED-EV-S. miR-146a-3p was predominantly expressed in BM-MSC-EV-S, whereas miR-205-5p exhibited comparable expression across SHED-EV-L, BM-MSC-EV-L, and BM-MSC-EV-S. Multiple let-7 family members, including let-7a-2-3p, let-7c-3p, and let-7f-1-3p, displayed relatively higher expression in SHED-EV-L and BM-MSC-EV-S. Together, these results suggest that both the tissue of origin and the culture phase critically influence the enrichment of miRNAs functionally linked to epithelial repair and fibrosis regulation.

## 3. Discussion

In this study, we observed that several regeneration- and fibrosis-related miRNAs exhibited distinct expression patterns depending on both the MSC source and the culture growth phase. Among these, miR-31-3p was consistently abundant in SHED-derived EVs, regardless of phase, and was also moderately expressed in UC-MSC-EV-L and BM-MSC-EV-L. miR-31-5p showed a clear phase-dependent increase in SHED-EV-S compared with SHED-EV-L. Members of the miR-99 family, particularly miR-99a-3p and miR-100-3p, were preferentially enriched in SHED-EV-S, whereas miR-146a-3p was most prominently expressed in BM-MSC-EV-S. miR-205-5p displayed comparable enrichment across SHED-EV-L, BM-MSC-EV-L, and BM-MSC-EV-S. Several let-7 family members were relatively more abundant in SHED-EV-L and BM-MSC-EV-S, while miR-21-5p remained at low levels in all conditions.

These differential expression profiles suggest potential phase-specific therapeutic implications for enhancing mucosal healing after urethral reconstruction. During the early postoperative (acute) phase, SHED-EV-L and BM-MSC-EV-S—with elevated levels of miR-31, let-7 family members, and miR-205—may accelerate epithelial closure and improve graft take while simultaneously exerting tumor-suppressive effects. By contrast, during the tissue maturation (chronic) phase, BM-MSC-EV-L and SHED-EV-S—enriched not only in miR-31 but also in the miR-99 family (miR-99a/miR-100)—may support epithelial stabilization, regulate inflammatory responses, and limit fibrotic scar formation.

Quantitatively, EV production tended to decrease from the log phase to the stationary phase in most MSC types, except for UC-MSCs, which maintained or even slightly increased EV yield (Table 1). This finding highlights the tissue origin-dependent differences in EV secretion dynamics across different growth phases. These characteristics render UC-MSCs particularly advantageous for scalable EV production, especially in therapeutic settings that require large quantities of vesicles. Moreover, understanding how EV biogenesis is influenced by the growth phase of MSC cultures, particularly in relation to their tissue origin, may offer critical insights into optimizing cell culture conditions tailored to specific regenerative or anti-fibrotic applications

While oral mucosal graft (OMG) urethroplasty achieves high success in expert centers, access remains limited by procedural complexity. We therefore envision two complementary pathways for EVs: (i) as an adjunct to OMG in complex reconstructions at referral centers, to support acute epithelialization and temper fibroblast activation, and (ii) as an adjunct to endoscopic management (dilation or DVIU) deliverable through a standard cystoscope. Practical formats include intralesional/submucosal injection of an EV-laden, mucoadhesive gel immediately after the incision or dilation, transurethral instillation of a shear-thinning or in situ-gelling EV formulation to coat the treated segment, and short-term placement of an EV-releasing catheter to provide sustained local delivery during early healing. This pro-regenerative strategy targets the biological drivers of restenosis (incomplete re-epithelialization and exuberant fibrosis) while preserving a simple, scalable workflow that could broaden patient access. Head-to-head studies against current endoscopic modalities, including drug-coated balloons, will be needed; the present brief report is hypothesis-generating and does not establish clinical efficacy.

Despite these promising findings, several limitations of this study should be acknowledged. First, this brief report was conceived as a hypothesis-generating molecular profiling study. Accordingly, we focused on phase- and origin-dependent miRNA signatures of MSC-derived EVs and did not include functional assays. Our results should therefore be interpreted as phenotypic/molecular descriptions rather than evidence of causality. In a subsequent full-length study, we plan to test the translational hypotheses suggested here. Specifically, we will evaluate whether phase-biased EV miRNA cargo supports acute epithelialization/engraftment, using standardized in vitro assays and in vivo validation. Until such data are available, the present conclusions should be considered preliminary. Second, all EV isolations and characterizations were performed under standardized in vitro conditions, which may not fully reflect the in vivo tissue environment. Third, while we compared four clinically relevant MSC sources, other promising origins, such as placenta- or iPSC-derived MSCs, were not examined and warrant future investigation. Fourth, we adopted confluence-based operational criteria to standardize EV harvest across donors and tissue sources. Accordingly, the “-L” and “-S” labels should be interpreted as pre-confluent expansion and post-confluent plateau, respectively, rather than as precise cell-cycle designations.

Together, these findings underscore the feasibility of tailoring MSC-EV therapies not only by cell source but also by culture growth phase, specifically the log and stationary phases, to align with distinct stages of tissue repair. The results revealed growth phase- and source-specific miRNA profiles with unique regenerative and anti-fibrotic signatures, supporting the concept of a phase-matched, EV-based strategy to optimize urethral healing across a broader patient population. This approach may be particularly valuable in clinical contexts where access to formal reconstructive surgery is limited, providing biologically guided enhancement to conventional endoscopic procedures. This study offers preclinical support for the development of EV-based adjunctive therapies customized by MSC origin and culture phase to improve the integration and long-term viability of oral mucosal grafts in urethral reconstruction.

## 4. Materials and Methods

### 4.1. Cell Sources and Culture Conditions

Stem cells from human exfoliated deciduous teeth (SHED; Summit Pharmaceuticals International, Tokyo, Japan, ATCC^®^ CRL-3485™) and mesenchymal stromal cells (MSCs) derived from adipose tissue (AD), umbilical cord (UC), and bone marrow (BM) (all from Summit Pharmaceuticals International, ATCC^®^ PCS-500 series) were obtained from commercial suppliers. Cells were maintained at 37 °C in a humidified 5% CO_2_ atmosphere. SHEDs were seeded at 1500 cells/cm^2^ and cultured in a xeno-free medium supplemented with human serum (Takara Bio, Kusatsu, Japan, Cat. Y30001) until they reached ~5000 cells/cm^2^ over 36 h, representing the logarithmic (log) growth phase. The culture was then switched to an animal-origin-free (AOF) medium (Rohto Pharmaceutical, Osaka, Japan). AD-, UC-, and BM-MSCs were each seeded at 5000 cells/cm^2^ and transitioned to AOF medium 12 h after attachment.

### 4.2. Definition of Growth Phases and Conditioned Medium Collection

EV harvest was triggered by objective culture features rather than elapsed time, followed by a fixed CM collection window in AOF medium. Logarithmic (EV-L) cultures were defined as pre-confluent monolayers (40–70% confluence) showing ongoing expansion over the preceding 24 h (≥30% increase in viable cell counts per well) and viability ≥ 90%. Stationary (EV-S) cultures were defined as monolayers at ≥95% confluence maintained for 24–48 h with plateaued growth (≤10% change in viable counts over 24 h) and viability ≥ 90%. Once the above criteria were met, cultures were maintained in AOF and CM was collected over a 24 h window. For SHED, the switch from xeno-free medium with human serum to AOF was performed immediately prior to EV collection; AD-, UC-, and BM-MSCs were already maintained in AOF after attachment. Throughout, samples are denoted by source and phase (e.g., SHED-EV-L, BM-MSC-EV-S).

### 4.3. Extracellular Vesicle Isolation and Particle Characterization by Nanoparticle Tracking Analysis

Conditioned media (CM) were collected as described in Section 4.2 to capture EVs released during the log (−L) and stationary (−S) phases. CM were sequentially clarified by low-speed centrifugation (300× *g* for 10 min and 2000× *g* for 10 min) to remove cells and debris, followed by 0.22-µm filtration. Extracellular vesicles (EVs) were isolated using a polymer-based precipitation method (EXO Isolation Reagent Kit, Dojindo, Cat. EX10, Dojindo, Kumamoto, Japan) according to the manufacturer’s protocol. EV pellets were resuspended in sterile, 0.22-µm-filtered PBS and gently mixed on ice. To avoid repeated freeze–thaw cycles, resuspended EVs were aliquoted for particle analysis and RNA extraction. Throughout the study, samples are denoted by source and growth phase (e.g., SHED-EV-L, BM-MSC-EV-S).

Particle concentration and size distribution were determined by nanoparticle tracking analysis (NTA) on a NanoSight system (Malvern Instruments, Worcestershire, UK). For each biological replicate, three 60 s videos were acquired at room temperature with identical camera level and detection threshold across all conditions. Instrument performance was verified at each session using the vendor’s standard operating procedures.

### 4.4. EV RNA Extraction, Microarray-Based miRNA Profiling, and Data Processing

Total RNA, including small RNAs, was extracted from EV aliquots using a phenol-free, column-based method compatible with low-input samples, following the manufacturer’s instructions. RNA yield was quantified using fluorescence-based assays optimized for small RNAs.

Comprehensive miRNA expression profiling was performed with the human 3D-Gene microarray platform (Toray Industries, Tokyo, Japan) under xeno-free/serum-free collection conditions. Labeling, hybridization, washing, and scanning were carried out according to the manufacturer’s protocols. Raw intensity files were exported for downstream processing. Background correction and global median normalization were applied across arrays, and expression values were log_2_-transformed for visualization and comparative analyses.

For the global heatmap (Figure 1A), the top 50 miRNAs by median expression across all conditions were selected to depict overall distribution patterns. For the focused analysis (Figure 1B), we examined miRNAs with reported relevance to epithelial regeneration and fibrosis modulation: miR-31-3p/-5p, miR-21-5p, miR-205-5p, miR-99a-3p, miR-100-3p, miR-17-5p, miR-146a-3p, and selected let-7 family members (Table 2 for functional summaries and primary references). Heatmaps display median-centered, normalized values, with red indicating higher and blue indicating lower expression relative to the median.

## 5. Conclusions

Extracellular vesicles from mesenchymal stromal cells exhibit growth phase- and origin-specific miRNA profiles, supporting a strategy of tailoring EV selection by cell source and culture phase (log vs. stationary) to optimize urethral regeneration; however, functional validation is needed before clinical application.

## Figures and Tables

**Table 1:** Extracellular Vesicle (EV) Concentration from MSC Sources.

MSC Origin	Log Phase (particles/mL)	Stationary Phase (particles/mL)
SHED	1.97 × 10^9^	1.18 × 10^9^
AD-MSC	1.64 × 10^9^	1.10 × 10^9^
UC-MSC	1.39 × 10^9^	1.54 × 10^9^
BM-MSC	1.49 × 10^9^	4.18 × 10^8^

EV concentrations (particles/mL) were quantified using nanoparticle tracking analysis (NanoSight, Malvern Instruments, Worcestershire, UK). Abbreviations: MSC = mesenchymal stromal cell; SHED = stem cells from human exfoliated deciduous teeth; AD = adipose-derived; UC = umbilical cord-derived; BM = bone marrow-derived.

**Table 2:** Functional Overview of Selected miRNAs Related to Mucosal Regeneration and Fibrosis Modulation.

miRNA	Primary Function	Target Pathways/Molecules	Clinical Significance for Mucosal Grafting	Reference
**miR-31**	Promotes proliferation and migration	RhoA, FZD3, Wnt/β-catenin	Accelerates initial epithelial closure and graft integration	[10,11,12]
**miR-21**	Anti-apoptotic, pro-survival, anti-inflammatory	PTEN/AKT, TGF-β	Facilitates early tissue adaptation and inhibits fibrotic response	[13,14,15]
**miR-205**	Maintains epithelial phenotype, inhibits EMT	ZEB1/2, E-cadherin	Promotes epithelial integrity; downregulation is required for keratinocyte migration during re-epithelialization; potential therapeutic target in chronic wounds	[16]
**miR-99 family** **(miR-99a/miR-100)**	Regulates proliferation and metabolism	mTOR/IGF1R pathway	Maintains controlled epithelial proliferation and energy balance during early repair	[17,18,19]
**miR-17-5p**	Regulates epithelial–mesenchymal crosstalk; fibroblast and endothelial activity	TGF-β, Col1A1; angiogenic regulators	May support matrix remodeling, but excessive activity can enhance fibrosis; downregulation improves endothelial survival and angiogenesis	[20]
**miR-146a**	Anti-inflammatory, antioxidative, immune-suppressive	TRAF6, IRAK1	Resolves chronic inflammation, reduces oxidative stress, and facilitates transition from inflammatory to proliferative phase during wound healing	[21]
**let-7 family**	Controls cell cycle and differentiation; tumor suppression	RAS, HMGA2	Promotes epithelial stability and suppresses oncogenic transformation in vitro and in vivo	[22,23,24]

## Data Availability

The data presented in this study are available in this article.

## Abbreviations

The following abbreviations are used in this manuscript:

EV	Extracellular vesicle
MSC	Mesenchymal stromal cell
SHED	Stem cells from human exfoliated deciduous teeth
AD-MSC	Adipose-derived mesenchymal stromal cell
UC-MSC	Umbilical cord-derived mesenchymal stromal cell
BM-MSC	Bone marrow-derived mesenchymal stromal cell
miRNA	MicroRNA
DVIU	Direct visual internal urethrotomy
NTA	Nanoparticle tracking analysis
AOF	Animal-origin-free (medium)
OMG	Oral Mucosal Graft
